# Relationship between biological rhythm dysregulation and suicidal ideation in patients with major depressive disorder

**DOI:** 10.1186/s12888-024-05528-2

**Published:** 2024-01-31

**Authors:** Dan Liu, Min Zhang, Lei Ding, Jia Huang, Yun Wang, Yousong Su, Zheng Chen, Yiyun Cai, Shen He, Daihui Peng

**Affiliations:** Shanghai Mental Health Center, Shanghai Jiao Tong University School of Medicine, 600 South Wan Ping Road, 200030 Shanghai, People’s Republic of China

**Keywords:** Major depressive disorder, Biological rhythm, Suicide ideation, Biological rhythms interview of assessment in neuropsychiatry

## Abstract

**Background:**

Although the disturbance of circadian rhythms represents a significant clinical feature of major depressive disorder (MDD), the relationship between biological rhythm disturbances and the severity of suicidal ideation in individuals with MDD remains unclear. We aimed to explore the characteristics of different biological rhythm dimensions in MDD and their association with the severity of depressive symptoms and suicidal ideation.

**Methods:**

A total of 50 MDD patients and 50 healthy controls were recruited and their general information was collected. The severity of depressive symptoms was assessed with the 17-item Hamilton Depression Rating Scale (HDRS_17_). The intensity of suicidal ideation was evaluated with the Beck Scale for Suicide Ideation (BSS). The Chinese version of the Biological Rhythms Interview of Assessment in Neuropsychiatry (BRIAN) scale was utilized to assess the participants’ biological rhythm dysregulation. Multiple logistic regression analysis was conducted to explore the relationship between biological rhythm and the risk of MDD. Multiple linear regression analysis was performed in the MDD group to investigate the relationship between different biological rhythm dimensions and suicide ideation.

**Results:**

Significant differences were observed between the MDD group and the control group in total BRIAN score (Z=-5.41, *P* < 0.001) as well as scores for each dimension. After adjusting for confounding factors, multiple logistic regression analysis revealed a significant association between total BRIAN score and the presence of MDD (OR = 1.20, 95% CI = 1.10–1.29, *P* < 0.001), as well as between scores in different BRIAN dimensions and the presence of MDD (activity: OR = 1.47, 95% CI = 1.24–1.74, *P* < 0.001; sleep: OR = 1.52, 95% CI = 1.28–1.79, *P* < 0.001; social: OR = 1.80, 95% CI = 1.32–2.46, *P* < 0.001; eating pattern: OR = 1.34, 95% CI = 1.12–1.60, *P* = 0.001). In patients with MDD, linear regression analysis demonstrated a positive relationship between BSS scores and BRIAN eating pattern scores (β = 0.34, *P* = 0.022), even after adjusting for demographic factors and the severity of depression.

**Conclusions:**

Patients with MDD exhibited significantly higher levels of dysregulation in all four biological rhythm dimensions compared to healthy controls and the degree of dysregulation was associated with the severity of depression. More importantly, dysregulation of eating pattern may increase the intensity of suicidal ideation in MDD, thus elevating the risk of suicide.

## Background

Circadian rhythms refer to the cyclic behavioral and physiological phenomena occurring within organisms, including the sleep-wake cycle, hormone secretion, feeding habits and energy metabolism [1]. The development of biological clocks is considered a biological strategy that has evolved in response to the daily changes caused by a constantly changing environment, thereby enhancing the organism’s ability to survive. However, the requirements of contemporary lifestyles, such as extended periods spent indoors without access to natural light and exposure to artificial light after sunset, have led to changes in daily behavior that go against the natural circadian rhythms. This disturbance in lifestyle could elevate the likelihood of developing various illnesses, including cancer, cardiovascular diseases, and metabolic disorders [2].

In addition to core symptoms such as persistent sadness, reduced interest, and decreased energy, patients with major depressive disorder (MDD) often experience disturbances in circadian rhythms. These disruptions manifest as irregular sleep-wake cycles, diurnal mood variations, seasonal fluctuations, changes in eating habits, and alterations in daily social functioning [3]. Studies have shown a close correlation between circadian rhythm disruptions and the onset, symptomatology, recurrence, and prognosis of MDD [4]. The disturbance of circadian rhythms represents a significant clinical feature of MDD [5].

Circadian clock genes are responsible for encoding proteins and RNA that participate in the control of circadian rhythms. Through intricate molecular regulatory networks, these genes interact to maintain an organism’s circadian rhythm. Studies have indicated that MDD patients show irregular timing of circadian gene expression and disturbed phase relationships among individual circadian genes [6]. One study demonstrated that the downregulation of expression in the crucial clock gene Bmal1 within the suprachiasmatic nucleus disrupts the typical circadian rhythm of corticosterone and diminishes the stress-induced elevation of corticosterone, thereby inducing depressive-like behavior in mice [7]. This finding provides new targets for the treatment of depression. Additionally, many antidepressant therapies, such as pharmacological treatments (e.g., agomelatine, ketamine), social rhythm therapy, and light therapy, directly impact circadian rhythms [8–10]. Hence, investigations on circadian rhythms hold significant importance both in understanding the pathology of MDD and in developing therapeutic interventions for its management.

Patients with MDD exhibit a heightened risk of suicide. Epidemiological data suggests that suicide events follow certain rhythmic patterns, especially circadian and seasonal patterns, with a higher frequency during the spring and summer compared to the autumn and winter and a distinct timing preference within different age groups [11, 12]. Previous research on the relationship between suicide and circadian rhythms in MDD has primarily focused on chronotype and seasonal patterns, indicating that patients with higher seasonality or evening-type tendencies exhibit increased suicidality [13]. However, a gap in the literature exists regarding the association between the degree of rhythm disruption itself and the intensity of suicidal ideation. Research in individuals with bipolar affective disorder suggests a relationship between the degree of rhythm disruption and the intensity of suicidal ideation [14, 15]. Therefore, we postulate that disturbances in circadian rhythms may be associated with the intensity of suicidal ideation in MDD.Previous clinical assessment scales evaluating biological rhythms have primarily concentrated on chronotype, such as the Morningness-Eveningness Questionnaire and the Composite Scale of Morningness [16, 17]. Chronotype refers to an individual’s inherent or genetically determined preference for specific times of day when they are most alert and active. It can be categorized into morning chronotypes (morning people or “larks”) and evening chronotypes (night owls or “owls”). Chronotype takes into account an individual’s preferences and tendencies for specific times of day and only constitutes a specialized facet of the broader construct of circadian rhythms [18]. Meanwhile, many questionnaires pertaining to circadian rhythms predominantly center on the sleep dimension, such as the Pittsburgh Sleep Quality Index, which solely allows for the assessment of sleep quality and its influence on daily functioning, thereby overlooking other aspects [19]. Increasing evidence indicates that social and activity rhythms associated with daily work, social engagement, and recreational activities are also integral components of the circadian system [10]. These rhythms have the potential to impact the secretion of hormones and neurotransmitters, thus potentially mediating the occurrence of MDD.

The Biological Rhythms Interview of Assessment in Neuropsychiatry (BRIAN) is a comprehensive clinical evaluation tool that assesses the biological rhythms of mood disorder patients across various dimensions, including sleep, social rhythms, activity, and eating pattern [20]. This scale has been translated into multiple languages and has been widely used in clinical settings in countries such as South Korea, Japan, and Italy [21–23]. Research on the Chinese population with MDD has found that the Chinese version of the scale demonstrates good reliability and validity [24]. This study also reported that individuals with MDD in China exhibit a more significant degree of biological rhythm disturbances than healthy controls. However, the relationship between biological rhythm disturbances and the severity of depressive symptoms in the Chinese population remains unclear. Also, as mentioned earlier, there is a lack of research exploring the relationship between biological rhythm disturbances and the severity of suicidal ideation in individuals with MDD. Therefore, this study aims to explore the relationship between sleep, social, activity, and eating rhythm disturbances and the severity of depressive symptoms and suicide ideation in MDD.

## Methods

### Participants

Between October 2022 and May 2023, a group of 50 individuals aged 18 to 65 years, diagnosed with MDD based on clinical evaluation by a specialized physician using the Diagnostic and Statistical Manual of Mental Disorders, Fifth Edition (DSM-5) criteria, were recruited from the Shanghai Mental Health Center. Additionally, 50 healthy controls, matched in age and sex, were selected by consecutive sampling within the same center. These control subjects included employees, graduate students, and their acquaintances, all of whom were chosen after exclusion of any psychiatric illness history. The study was carried out in full compliance with the principles of the Declaration of Helsinki and obtained approval from the institutional review board of the Shanghai Mental Health Center (ethics approval number: 2019-17C1). Prior to participation, all individuals willingly agreed to take part and provided written informed consent.

### Assessments

Demographic information was collected. The severity of depressive symptoms is assessed with the 17-item Hamilton Depression Rating Scale (HDRS_17_) [25]. The HDRS_17_ comprises 17 items, with each item being scored on a scale ranging from 0 to 2 or 0 to 4. The total score on the scale falls within the range of 0 to 52, where higher scores are indicative of greater severity of depressive symptoms. The Chinese version of the scale showed good reliability and validity, with a Cronbach’s alpha of 0.714 [26]. The Beck Scale for Suicide Ideation (BSS) is employed to evaluate suicidal ideation. It comprises a total of 19 items and requires respondents to provide answers based on their experiences over the past week. Utilizing a 3-point rating system, the total score ranges from 0 to 38 points, with higher scores indicating stronger suicidal thoughts [27]. The Chinese version of the scale has been demonstrated to possess robust reliability and validity, with a Cronbach’s alpha coefficient between 0.60 and 0.89 among different settings [28]. The Chinese version of the Biological Rhythms Interview of Assessment in Neuropsychiatry (BRIAN) is utilized to evaluate biological rhythm disturbances. This scale consists of 21 items that comprehensively evaluate the participants’ biological rhythms across four dimensions: sleep (BRIAN-Sleep, items 1–5), activities (BRIAN-Activity, items 6–10), social rhythms (BRIAN-Social, items 11–14), and eating patterns (BRIAN-Eating pattern, items 15–18). Additionally, items 19–21 were used to assess the dominant biological rhythm and were not included. All items were scored on a 1–4 scale, with a total score ranging from 18 to 72, where higher scores indicate a higher level of biological rhythm disturbances. The Chinese version of the scale (C-BRIAN) demonstrated good reliability and validity in previous studies conducted by our research group, with a overall Cronbach’s α value of 0.898 [24].

### Statistical analyses

Statistical analysis was performed using SPSS 24.0. Categorical variables such as gender were described using frequency (percentage) and intergroup comparisons were conducted using the chi-square test. The Kolmogorov-Smirnov test was used to check normal distribution. Body mass index (BMI), which followed a normal distribution, was described using the mean ± standard deviation (SD), and intergroup comparisons were performed using independent samples t tests. Age, BSS scores, HDRS_17_ scores, BRIAN total score, and scores of individual scales that did not follow a normal distribution were described using median (lower quartile, upper quartile) [M (QL, QU)], and intergroup comparisons were conducted with the Mann-Whitney U test.

The correlation between biological rhythm, severity of depressive symptoms, and severity of suicidal ideation was analyzed using Spearman’s rank correlation analysis. This analysis found moderately high correlations between the BRIAN subscale scores, with coefficients ranging from 0.46 to 0.73 (all p-values significant). Additionally, the Variance Inflation Factor (VIF) was also employed to assess collinearity between the different dimensions. When placing the various dimensions into the same model, the VIF values for two BRIAN subscales exceeded 2.5, which is generally indicative of considerable collinearity [29]. Therefore, we determined there was evidence of collinearity between the BRIAN dimension variables. Univariate logistic regression analyses were conducted with the degree of rhythm disruption in each BRIAN dimension as the independent variable and diagnosis of MDD as the dependent variable. Due to the strong collinearity among the different dimensions of the BRIAN scale demonstrated by Spearman’s rank correlation analysis, we entered each BRIAN dimension along with three demographic confounding factors into separate multivariate logistic regression models using the enter method. This allowed us to assess the independent predictive utility of each subscale while accounting for the demographic confounding factors. The multivariate logistic regression models were constructed with diagnosis of MDD as the dependent variable. The independent variables in each model included three demographic confounding factors (gender, age, and BMI) plus one of the BRIAN dimension subscales.

To investigate the relationship between the degree of biological rhythm disturbances and the severity of suicidal ideation in MDD, a univariate linear regression analysis was first conducted. The BSS score was set as the dependent variable, and each BRIAN dimension subscale score was set as an independent variable in separate models. This allowed identification of the rhythm dimensions that were significantly associated with suicidal ideation. Subsequently, using the enter method, multivariate linear regression models were constructed with BSS score as the dependent variable. The independent variables entered into each model included each significant dimension identified in the univariate analyses along with confounding factors of age, BMI, gender and HDRS_17_ score. This approach enabled assessment of the effects of biological rhythm disturbances on suicidal ideation severity while adjusting for potential confounders.

To diagnose multicollinearity among the variables in the constructed models, Spearman correlation coefficients and VIF were utilized. Multicollinearity was considered absent if all VIF values were under 2.5 and correlation coefficients were below 0.5 [29, 30]. A significance level of *p* < 0.05 was considered statistically significant.

## Results

### Participant characteristics

A total of 50 MDD patients and 50 healthy controls were included (Table 1). There were no statistically significant differences in terms of age (Z=-0.90, *P* = 0.367), gender (χ^2^ = 0.04, *P* = 0.836), or BMI (t=-0.78, *P* = 0.440) between the two groups.

The median HDRS_17_ scores were 15.00 (10.00, 18.25) and 2.00 (0.00, 3.00) for the MDD group and control group, respectively. There was a significant statistical difference between the two groups (Z=-7.52, *P* < 0.001). The median BSS score for the MDD group was 1.50 (0.00, 14.25), whereas it was 0.00 (0.00, 0.00) for the control group,and the difference remained statistically significant(Z=-4.74, *P* < 0.001)(Table 1).

**Table 1:** Characteristics of the study participants (*n* = 100)

characteristic	MDD(*n* = 50)	Health controls(*n* = 50)	χ2/t/Z	p value
gender,n(%)			0.04	0.836
male	18(36.00)	19(38.00)		
female	32(64.00)	31(62.00)		
Age,y, M (QL,QU)	26.50(20.75,46.00)	25.50(24.75,45.50)	-0.90	0.367
BMI,kg/m^2^ mean(SD)	22.84(4.22)	22.26(3.30)	-0.78	0.440
HDRS_17_ score, M (QL,QU)	15.00(10.00,18.25)	2.00(0.00,3.00)	-7.52	< 0.001
BSS score, M (QL,QU)	1.50(0.00,14.25)	0.00(0.00,0.00)	-4.74	< 0.001
BRIAN score, M (QL,QU)	41.00(26.75,45.25)	23.00(20.75,26.00)	-5.41	< 0.001

BRIAN = Biological Rhythm Interview Assessment in Neuropsychiatry;

HDRS_17_ = 17-item Hamilton Depression Rating Scale

BSS = Beck Scale for Suicide Ideation

Body mass index (BMI) was described as mean ± standard deviation (SD), and intergroup comparisons were performed using independent samples t tests.

Age, BSS scores, HDRS17 scores and BRIAN scores were described as median (lower quartile, upper quartile) [M (QL, QU)], and intergroup comparisons were conducted with the Mann-Whitney U test.

Gender was described as frequency (percentage) and intergroup comparison was conducted using the chi-square test.

The significance threshold was set at 0.05.

The BRIAN scale scores showed significant differences between the two groups both in the total score (Z=-5.41, *P* < 0.001) and the scores for sleep (Z=-5.14, *P* < 0.001), activity (Z=-5.11, *P* < 0.001), social (Z=-4.17, *P* < 0.001), and eating pattern (Z=-2.25, *P* = 0.025)(Table 2).

**Table 2:** Comparison of BRIAN domains between patients with MDD and healthy controls

BRIAN score	MDD(*n* = 50)	Health controls(*n* = 50)	Z	p-value
BRIAN total score	41.00(26.75,45.25)	23.00(20.75,26.00)	-5.41	<0.001
BRIAN-Sleep	13.00(8.50,16.00)	8.00(5.00,9.00)	-5.14	<0.001
BRIAN-Activity	11.50(6.00,14.25)	5.00(5.00,7.00)	-5.11	<0.001
BRIAN-Social	5.50(4.00,8.25)	4.00(4.00,4.25)	-4.17	<0.001
BRIAN-Eating pattern	7.00(4.00,10.00)	5.00(4.00,6.25)	-2.25	0.025

BRIAN = Biological Rhythm Interview Assessment in Neuropsychiatry;

BRIAN total score, and scores of individual scales were described as median (lower quartile, upper quartile) [M (QL, QU)], and intergroup comparisons were conducted with the Mann-Whitney U test

The significance threshold was set at 0.05.

Additionally, there were positive correlations among the scores of different dimensions in the BRIAN scale (Table 3).

**Table 3:** Spearman correlations among the scores of different dimensions of the BRIAN scale

	BRIAN-Sleep	BRIAN-Activity	BRIAN-Social	BRIAN-Eating pattern
BRIAN-Sleep	1.000			
BRIAN-Activity	0.725^**^	1.000		
BRIAN-Social	0.598^**^	0.621^**^	1.000	
BRIAN-Eating pattern	0.472^**^	0.334^**^	0.460^**^	1.000

BRIAN = Biological Rhythm Interview Assessment in Neuropsychiatry;

Correlations significant at ** 0.01 (two-tailed)

### Biological rhythm disturbances were associated with an elevated risk of developing MDD

Univariate logistic regression analysis revealed statistically significant associations between the total score and individual dimension scores of BRIAN and MDD. Furthermore, after adjusting for confounding factors such as gender, age, and BMI in a multivariate logistic regression analysis, the associations remained statistically significant (Total BRIAN score: OR = 1.20; BRIAN-Activity: OR = 1.47; BRIAN-Sleep: OR = 1.52; BRIAN-Social: OR = 1.80; BRIAN-Eating pattern: OR = 1.34)(Table 4).

**Table 4:** Logistic regression analyses of the effects of biological rhythms on MDD

	Univariate logistic regression OR(95%CI)	p value	multivariate logistic regression OR(95%CI)	p value
BRIAN-Sleep	1.44(1.24,1.68)	< 0.001	1.52 (1.28,1.79)	< 0.001
BRIAN-Activity	1.45(1.24,1.71)	< 0.001	1.47 (1.24,1.74)	< 0.001
BRIAN-Social	1.66(1.26,2.20)	< 0.001	1.80 (1.32,2.46)	< 0.001
BRIAN-Eating pattern	1.27(1.08,1.48)	0.003	1.34 (1.12,1.60)	0.001
BRIAN total score	1.16 (1.10,1.24)	< 0.001	1.20 (1.10,1.29)	< 0.001

BRIAN = Biological Rhythm Interview Assessment in Neuropsychiatry;

OR = odds ratio; 95% CI = 95% confidence interval

### Elevated levels of biological rhythm disturbances were associated with increased severity of depressive symptoms in MDD

**Fig. 1:**
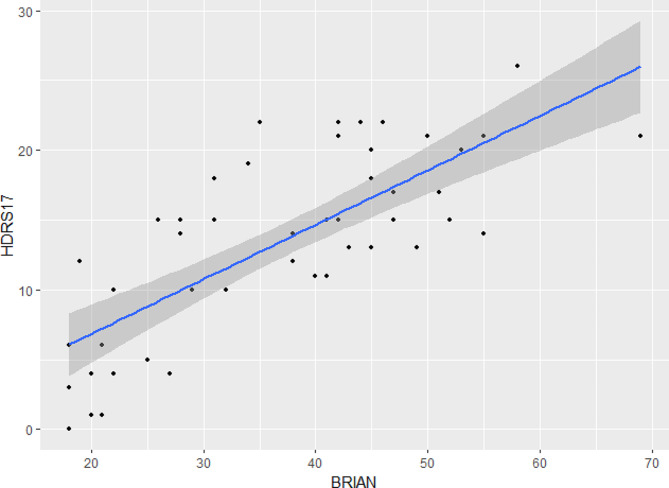
Scatter plot of BRIAN total score and HDRS_17_ score in patients with MDD. BRIAN = Biological Rhythm Interview Assessment in Neuropsychiatry; HDRS17 = 17-item Hamilton Depression Rating Scale. The blue line represents the linear regression fit, and the shaded area depicts the confidence interval for the regression line

**Fig. 2:**
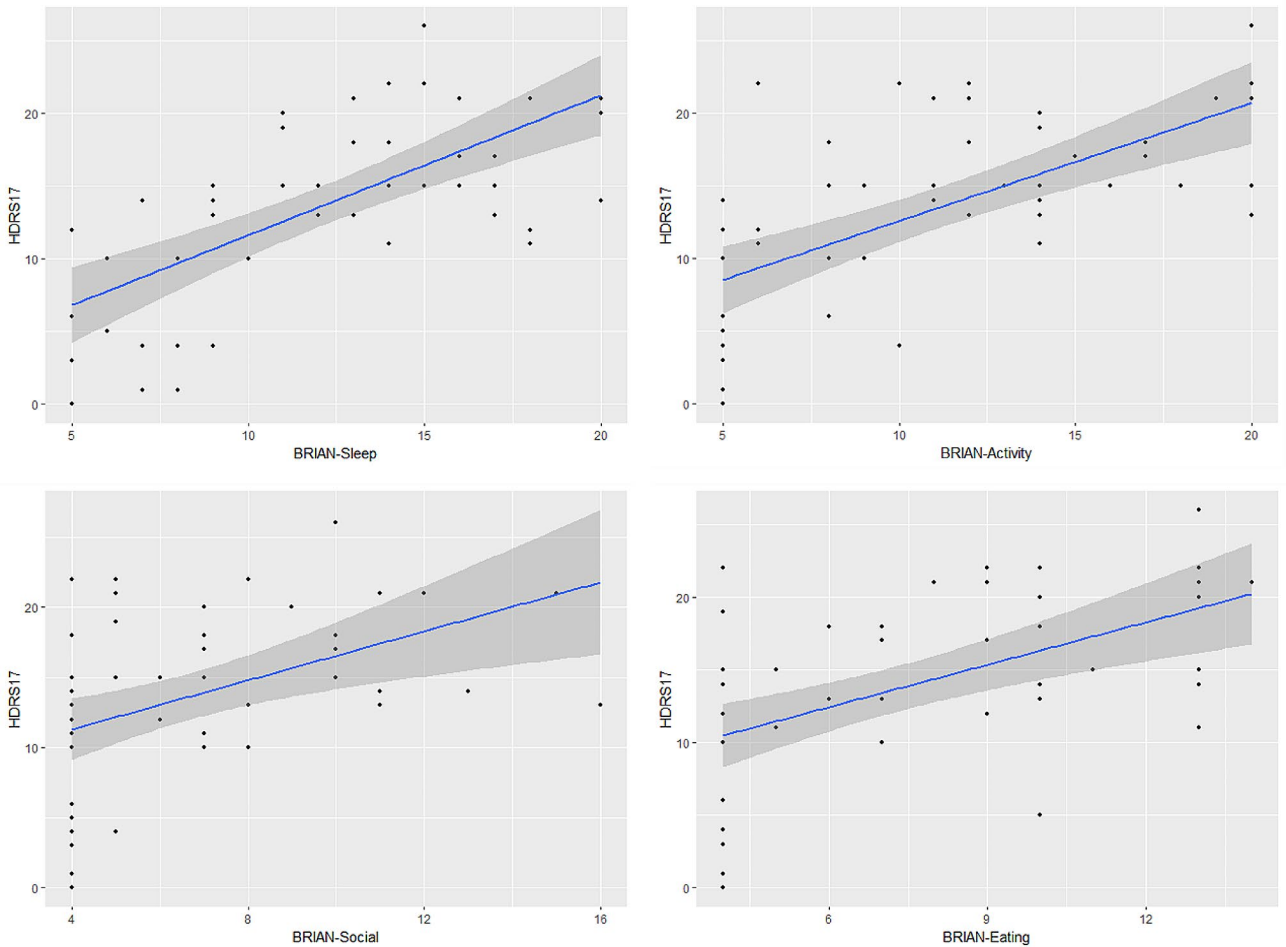
Scatter plots of different BRIAN domains and HDRS_17_ score in patients with MDD. BRIAN = Biological Rhythm Interview Assessment in Neuropsychiatry; HDRS_17_ = 17-item Hamilton Depression Rating Scale. The blue line represents the linear regression fit, and the shaded area depicts the confidence interval for the regression line

Spearman correlation analysis revealed a significant positive correlation between the severity of depressive symptoms in MDD and the scores of each dimension, as well as the total score, of the BRIAN scale(Table 5). Additionally, the scatter plot also illustrated that patients with higher levels of disturbance across dimensions of circadian rhythms tended to have increased severity of depressive symptoms (Figs. 1 and 2).

**Table 5:** Spearman correlations between BRIAN domains and HDRS_17_ scores in patients with MDD

	HDRS_17_ score	BRIAN-Sleep	BRIAN-Activity	BRIAN-Social	BRIAN-Eating pattern	BRIAN total score
HDRS_17_ score	1.000					
BRIAN-Sleep	0.629^**^	1.000				
BRIAN-Activity	0.633^**^	0.654^**^	1.000			
BRIAN-Social	0.466^**^	0.513^**^	0.506^**^	1.000		
BRIAN-Eating pattern	0.488^**^	0.499^**^	0.242	0.493^**^	1.000	
BRIAN total score	0.713^**^	0.833^**^	0.826^**^	0.759^**^	0.653^**^	1.000

BRIAN = Biological Rhythm Interview Assessment in Neuropsychiatry;

HDRS_17_ = 17-item Hamilton Depression Rating Scale

Correlations significant at ** 0.01 (two-tailed)

### An elevated degree of disruption in eating pattern rhythms increased the intensity of suicidal ideation

Univariate linear regression analysis with suicidal ideation intensity as the dependent variable revealed significant positive correlations between the HDRS_17_ score (β = 0.56, *P* < 0.001), BRIAN eating pattern score (β = 0.57, *P* < 0.001), and BSS score in MDD. Furthermore, we conducted a multivariate linear regression analysis with BSS score as the dependent variable and HDRS_17_ score, BRIAN eating pattern score, gender, age, and BMI as independent variables. The results demonstrated that the severity of depressive symptoms (β = 0.34, *P* = 0.014) and disruption in eating pattern rhythms (β = 0.34, *P* = 0.022) significantly increase the level of suicidal ideation in patients with MDD after adjusting for confounding factors. This model accounted for 37.5% of the variation in the severity of suicidal ideation (F = 6.891, *P* < 0.001)(Table 6), with VIF values below the threshold of 2.5.

**Table 6:** Linear regression analyses of BSS scores in MDD patients

	Univariate linear regression B(SE)	β	t	p value	multivariate linear regression B(SE)	β	t	p value
BRIAN-Eating pattern	1.46(0.30)	0.57	4.85	< 0.001	0.86(0.36)	0.34	2.37	0.022
HDRS_17_ score	0.76(0.16)	0.56	4.62	< 0.001	0.47(0.18)	0. 34	2.55	0.014

BRIAN = Biological Rhythm Interview Assessment in Neuropsychiatry;

HDRS_17_ = 17-item Hamilton Depression Rating Scale

B = unstandardized beta coefficient; β = standardized beta coefficient; SE = standard error

## Discussion

This study demonstrated that MDD patients exhibit significantly higher scores in all four dimensions and the total score of the biological rhythm assessment compared to healthy controls, indicating a higher degree of biological rhythm disturbances. Consistent with our findings, a study from Brazil in 2017 found that compared to healthy controls, MDD patients present significantly higher levels of biological rhythm disturbances among the 18–24 age group [31]. Additionally, a study by Ozcelik et al. in 2020 revealed significantly higher levels of biological rhythm disturbances in Turkish MDD patients than in healthy controls [32]. The findings of this study in the Chinese population align with these related international studies.

This study demonstrated that an increased level of circadian rhythm disruption may elevate the risk of MDD. Furthermore, the severity of depression was significantly positively correlated with the degree of disruption in each dimension of the circadian rhythm in MDD.

A study involving accelerometer data from over 90,000 participants revealed that a low relative amplitude, which measures the extent of disruption in circadian rhythmicity of rest-activity cycles, is linked to a higher likelihood of MDD [33]. This finding supports a robust association between circadian disruption and the risk of MDD, consistent with the results of our study. Previous studies focusing on the relationship between sleep rhythms and depression have frequently demonstrated associations between characteristics and disruptions in sleep rhythms and the severity of depressive symptoms, as well as the risk of MDD onset [31, 32, 34]. For example, one study indicated that compared to individuals with a morning preference, individuals with an evening preference experience more severe depressive symptoms among those with MDD [35]. In 2021, a Mendelian randomization study using summary-level genetic associations with diurnal preference and MDD revealed that individuals with a morning diurnal preference have a decreased risk of MDD [36]. Additionally, a study found that the addition of modafinil, an arousal-promoting agent, improves daytime wakefulness and the severity of depression symptoms in MDD patients who respond partially to selective serotonin reuptake inhibitors, highlighting the significant role of sleep rhythms in depression [37].

In the dimension of activity rhythms, relevant meta-analyses indicate that low levels of physical activity increase the risk of MDD [38]. Conversely, regular physical activity has been shown to improve depressive symptoms [39]. A cross-sectional analysis involving 1800 samples from the National Health and Nutrition Examination Survey (NHANES) demonstrated that disruption in activity patterns was associated with more than twice the odds of clinically significant depression symptoms [40]. The relationship between physical activity and depression is likely bidirectional. On the one hand, mood disturbances may result in reduced motivation, altered reward-seeking behavior, or impaired concentration/organization, which theoretically could lead to disrupted activity patterns. On the other hand, physical activity can regulate pathways involved in the onset of depression, such as oxidative stress pathways, monoamine neurotransmitters, and the HPA axis, thereby modulating depressive symptoms. Furthermore, engagement in physical activity can improve depressive symptoms by enhancing self-efficacy and increasing social support [41]. These findings are consistent with the results of our study.

Furthermore, the results indicated that social rhythm disruption may increase the risk of MDD and the severity of its symptoms. A related study demonstrated that individuals with MDD exhibit more disrupted social rhythms than healthy controls, and within individuals with MDD, the severity of depressive symptoms is positively correlated with the level of social rhythm disruption [42], consistent with the findings of this study. However, the study mentioned above included only 11 inpatient MDD patients and 19 healthy controls, and the assessment tool used to evaluate social rhythms primarily focused on the daily schedules of inpatient individuals. It should be noted that the daily schedules of inpatients are highly structured, and thus the influence of external factors on social rhythms cannot be ruled out. Currently, research exploring the importance of social rhythms in affective disorders has predominantly focused on bipolar affective disorder, with limited studies on MDD. Social rhythm therapy (SRT), based on the social rhythm hypothesis of depression, has been applied in clinical treatment for bipolar affective disorder, highlighting the significance of social rhythms in affective disorders [10]. However, the effectiveness of SRT in depression still requires further exploration.

Our study demonstrated that irregular eating patterns may increase the risk of MDD and the severity of its symptoms. Disrupted eating patterns may lead to depressive symptoms by impacting metabolism and subsequently the HPA axis, immune inflammation, and neuroendocrine regulatory factors [42]. In addition to the quantity of intake, meal timing is also an important component of circadian rhythms. One possible reason meal timing serves as an important zeitgeber (external environmental cue that sets the body’s circadian “clock”) is that behavioral rhythms, such as physical activity, meal timing, and sleep/wake cycles, must be coordinated with internal biological rhythms, including hormone fluctuations such as melatonin and body temperature. Food restriction is a potent zeitgeber that may cause desynchronization between peripheral and central clocks, thereby exacerbating depression severity [32]. However, a previous related study found no association between irregular eating patterns and depression severity, which contrasts with the findings of this study, so the relationship warrants further investigation [16].

Previous studies have demonstrated that circadian disruption significantly increases the intensity of suicidal ideation in bipolar affective disorder [15]. However, the relationship between circadian disruption and suicidal ideation in MDD remains unclear. The results of this study indicated that among the four domains of biological rhythms assessed, eating rhythm disruption was significantly associated with more severe suicidal ideation in MDD patients. Although MDD patients commonly exhibit eating-related symptoms such as decreased appetite, binge eating, and emotional eating, no studies to date have examined the relationship between irregular eating patterns and suicidal ideation in MDD. The eating rhythm domain of the BRIAN scale mainly evaluates the ability to maintain regular eating patterns, assessing aspects such as timing, frequency, and quantity of meals and dependence on stimulating foods [20].

To our knowledge, this is the first study to report that irregular eating rhythms were significantly associated with more intense suicidal ideation after adjusting for demographic factors and depression severity, while disruptions in other domains were not.

A previous large US study of 71,712 individuals found that subthreshold eating disorder symptoms increase adolescent suicide risk even in the general population [43], suggesting a link between eating patterns and suicide risk. A study of 817 community participants with MDD showed that suicide attempts were associated with binge eating symptoms after adjusting for other suicidality factors [44]. We speculate that irregular eating rhythms and suicide may share common underlying mechanisms. The biological mechanisms of suicide involve many factors such as the HPA axis, hormone metabolism, and fatty acid metabolism [45]. Studies show that suicide attempters have greater HPA axis dysregulation [46]. Additionally, a large study of 1,600 people revealed that an SD decrease in a polyunsaturated fatty acid is associated with a 14% increase in suicide risk [47]. Eating rhythm disruptions can cause abnormal secretion of hormones such as insulin, leptin, and cortisol [48]. Animal studies also show altered circadian gene expression with eating rhythm disruptions, causing metabolic changes such as increased body weight and fat, enlarged adipocytes, and reduced circulating polyunsaturated fatty acids [1]. Psychologically, a 2021 study found that perceived burdensomeness mediated between weight stigma and suicide risk [49]. Since the BRIAN scale does not assess psychological mechanisms, the psychological links between eating rhythms and suicide warrant further study.

Our study had several limitations. Firstly, due to the cross-sectional nature of the research, our ability was restricted to assessing correlations rather than causal relationships. Consequently, the underlying mechanisms governing these associations remain enigmatic and necessitate further investigation. Future investigations should contemplate the adoption of longitudinal study design to facilitate the establishment of causal relationships. Furthermore, it is essential to note that our study featured a small-sample single-center design, with all participants being selected by consecutive sampling within the same center, which may have introduced selection bias. To enhance the representativeness of findings, future studies should consider expanding the sample size and including patients from different centers across various regions in China. Meanwhile, another significant limitation lies in the sole reliance on subjective self-reports to evaluate biological rhythm disruptions, which may introduce individual biases. The integration of objective measurements could serve to mitigate this limitation and strengthen the validity of the data by minimizing the influence of personal subjectivity in the assessment.

## Conclusions

In summary, the results of this study demonstrated that MDD patients exhibit significant circadian disruption in the domains of sleep, activity, social, and eating rhythms. Furthermore, the extent of circadian disruption was positively correlated with depression severity. More importantly, our study revealed that greater eating rhythm irregularity is significantly associated with more intense suicidal ideation in MDD patients. This suggests that the assessment of eating rhythms should be emphasized in clinical suicide risk evaluations. Targeting eating patterns may represent a novel intervention approach for reducing suicide ideation and improving patient outcomes.

## Data Availability

The datasets used and/or analyzed during the current study are available from the corresponding author on reasonable request.

## Abbreviations

MDD: Major depressive disorder
HDRS_17_: 17-item Hamilton Depression Rating Scale
BSS: Beck Scale for Suicide Ideation
BRIAN: Biological Rhythms Interview of Assessment in Neuropsychiatry
